# Aerodynamic drag of modern soccer balls

**DOI:** 10.1186/2193-1801-2-171

**Published:** 2013-04-19

**Authors:** Takeshi Asai, Kazuya Seo

**Affiliations:** Institute of Health and Sport Sciences, B207, University of Tsukuba, Tsukuba, 305-8574 Japan; Yamagata University, Koshirakawa 1-4-12, Yamagata, 990-8560 Japan

**Keywords:** Aerodynamic, Drag, Ball, Soccer, Critical reynolds number, Fright trajectory, Sport

## Abstract

Soccer balls such as the Adidas Roteiro that have been used in soccer tournaments thus far had 32 pentagonal and hexagonal panels. Recently, the Adidas Teamgeist II and Adidas Jabulani, respectively having 14 and 8 panels, have been used at tournaments; the aerodynamic characteristics of these balls have not yet been verified. Now, the Adidas Tango 12, having 32 panels, has been developed for use at tournaments; therefore, it is necessary to understand its aerodynamic characteristics. Through a wind tunnel test and ball trajectory simulations, this study shows that the aerodynamic resistance of the new 32-panel soccer ball is larger in the high-speed region and lower in the middle-speed region than that of the previous 14- and 8-panel balls. The critical Reynolds number of the Roteiro, Teamgeist II, Jabulani, and Tango 12 was ~2.2 × 10^5^ (drag coefficient, *C*_*d*_ ≈ 0.12), ~2.8 × 10^5^ (*C*_*d*_ ≈ 0.13), ~3.3 × 10^5^ (*C*_*d*_ ≈ 0.13), and ~2.4 × 10^5^ (*C*_*d*_ ≈ 0.15), respectively. The flight trajectory simulation suggested that the Tango 12, one of the newest soccer balls, has less air resistance in the medium-speed region than the Jabulani and can thus easily acquire large initial velocity in this region. It is considered that the critical Reynolds number of a soccer ball, as considered within the scope of this experiment, depends on the extended total distance of the panel bonds rather than the small designs on the panel surfaces.

**Electronic supplementary material:**

The online version of this article (doi:10.1186/2193-1801-2-171) contains supplementary material, which is available to authorized users.

## Introduction

Following Thompson’s (1910) pioneering study of golf balls, studies of the aerodynamic characteristics of golf balls have focused on their drag coefficient (*C*_*d*_) values and their dimpled shapes (Bearman and Harvey 1976; Davies 1949; Smits and Ogg 2004). Similarly, other studies have investigated the flight of cricket balls (Mehta et al. 1983), baseballs (Watts and Sawyer 1975; Watts and Ferrer 1987; LeRoy et al. 2001; Nathan et al. 2008), tennis balls (Štěpánek 1988; Zayas 1986), and volleyballs (Wei et al. 1988); many of these have been reviewed by Mehta (1985). Previous studies of the aerodynamic characteristics of soccer balls have focused on traditional 32-panel balls such as the Adidas Roteiro, where each panel is a pentagon or a hexagon (Bray and Kerwin 2003; Asai et al. 2007; Goff and Carré 2009). In recent times, though, balls featuring a reduced number of panels have been used at major tournaments. For example, the Adidas Teamgeist II, the official match ball of the 2008 Beijing Olympic Games, has 14 panels, and the Adidas Jabulani, the official match ball of the South Africa 2010 FIFA World Cup, has only 8 panels. Despite this trend toward fewer panels (Asai et al. 2007), few studies (Asai and Kamemoto 2011) have focused on the aerodynamic characteristics of these balls. Having said that, the Adidas Tango 12, the new soccer ball used at the UEFA Euro 2012 and the 2012 London Olympics, has 32 panels having a new shape and a curved design. Therefore, it is necessary to understand the aerodynamic characteristics of this new ball.

In this study, we conducted a steady-state analysis of the newest soccer ball—the Adidas Tango 12 (32 panels)—and conventional soccer balls—the Adidas Roteiro (32 panels), Adidas Teamgeist II (14 panels), and Adidas Jabulani (8 panels)—through a wind tunnel experiment, and we clarified the drag coefficient and critical Reynolds number. A simple 2D flight trajectory simulation was conducted based on the drag coefficient, and the effects of the drag characteristics on the flight distance and flight trajectory were examined. The relationship between the critical Reynolds number and the extended total distances of the panel bonds of the soccer balls was examined, and the two were shown to have a high degree of correlation.

## Methods

### Wind tunnel test

**Figure 1:**
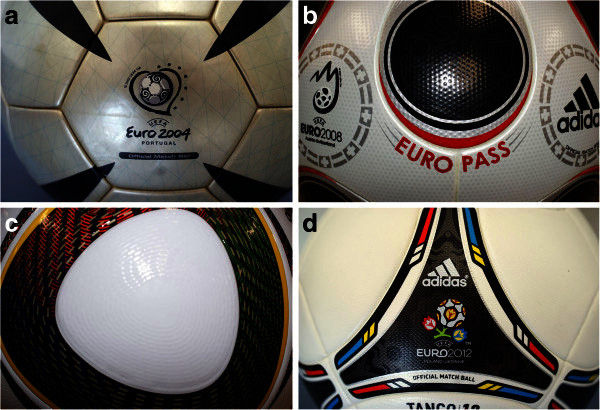
**Photographs of soccer balls.** (**a**) Adidas Roteiro: smooth surface with 32 pentagonal and hexagonal panels. (**b**) Adidas Teamgeist II: small protuberances with 14 panels. (**c**) Adidas Jabulani: small ridges or protrusions with 8 panels. (**d**) Adidas Tango 12: small grip texture with 32 modified panels.

We measured the aerodynamic forces acting on different types of balls in a low-speed wind tunnel having a 0.7 m × 0.7 m rectangular cross section (turbulence level: ≤1%). Four full-sized official FIFA soccer balls were tested: the conventional balls—the Adidas Roteiro (smooth surface with 32 pentagonal and hexagonal panels, used at UEFA Euro 2004), the Adidas Teamgeist II (small protuberances with 14 panels, used at the 2008 Beijing Olympic Games), and the Adidas Jabulani (small ridges or protrusions with 8 panels, used at the South Africa 2010 FIFA World Cup)—and the newly designed ball—the Adidas Tango 12 (small grip texture with 32 panels, used at UEFA Euro 2012 and the 2012 London Olympic Games) (Figure 1).

**Figure 2:**
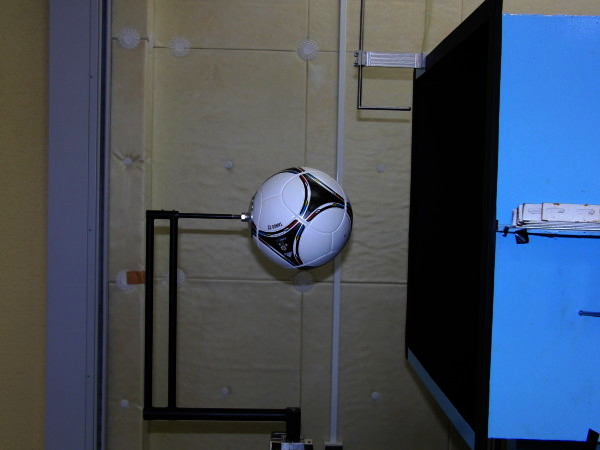
**Experimental setup with wind tunnel.**

Each soccer ball was attached to a stainless steel rod (Figure 2). In the wind tunnel experiment, the position of the support rod relative to the bluff body is important; therefore, we had to select an appropriate support method. In the experiment, we provided support from the rear (Achenbach, 1972), which we considered to have a comparatively smaller effect on peeling off of the boundary layer at the ball’s surface. We also measured the aerodynamic forces acting on the ball’s support without the ball. (The ball’s support does not make contact with the dummy ball.) This value was subtracted from the aerodynamic forces acting on the ball with the support. Data were acquired over a period of 8.192 s using a three-component strut-type balance (LMC-3531-50NS; Nissho Electric Works Co., Ltd.), and they were recorded on a personal computer using an A/D converter board (sampling rate: 1000/s). Each ball was set to be geometrically symmetrical; therefore, the ball panels were asymmetrical in the vertical direction.

The aerodynamic forces were measured at wind speeds, *U*, of 7–30 m/s. The force acting in the direction opposite to that of the wind (drag *D*) was calculated from the experimental data collected under different conditions. The measured aerodynamic forces were then used to calculate *C*_*d*_ using the following equation:1

Here, ρ is the density of air (1.2 kg/m^3^); *U*, the flow velocity (m/s); and *A*, the projected area (m^2^) of the soccer ball.

### Ball trajectory simulation

We conducted a simple 2D flight simulation to compare the effects of the drag coefficients of the Jabulani and the Tango 12 on their flight distance and flight trajectory (Goff and Carré 2009). The occurrence of irregular and unsteady Asai and Kamemoto 2011 flying with no spin or a low-speed spin (Asai and Kamemoto 2011). However, because this study focused on the relationship between the constant resistance of the ball and its flight trajectory, knuckle effects were ignored in the trajectory simulation. In the trajectory simulation we estimated the drag coefficient with respect to the Reynolds number using a cubic curve to calculate the two-dimensional coordinates of the ball. Therefore, we omitted the lift and side forces acting on the ball. We considered the effect of buoyancy on the flight trajectory to be negligible compared to the effect of drag; therefore, we omitted the buoyancy from our calculations. By using the relationship between the Reynolds number and the drag coefficient, which were measured in the wind tunnel experiment, we calculated the initial ball velocities of the two-dimensional flight trajectory to be 17 and 28 m/s, respectively; the ball was launched at an angle of 25° in both cases. We also computed the two-dimensional flight trajectories of the Jabulani (initial speed: 17 and 28 m/s) and the Tango 12 (initial speed: 17.4 and 28.7 m/s) under the condition of ball impacts having the same impulse (17 m/s: 7.45 kg/s; 28 m/s: 12.26 kg/s); in doing so, we considered the difference in mass of the Jabulani (0.438 kg) and the Tango 12 (0.428 kg).

### Extended total distances of panel bonds

**Figure 3:**
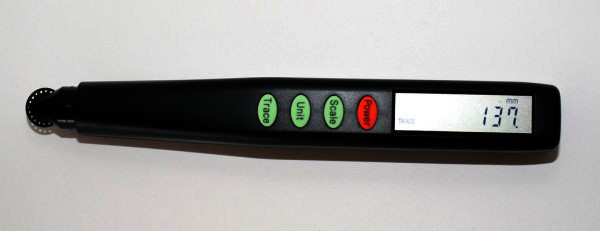
**Example photo of the curvimeter for measuring extended total distances.**

As an index of the surface roughness of the ball, we measured the extended total distances of the panel bonds using a curvimeter (Concurve 10; KOIZUMI Sokki Mfg. Co., Ltd.) (Figure 3).

## Results

### Drag force in wind tunnel test

**Figure 4:**
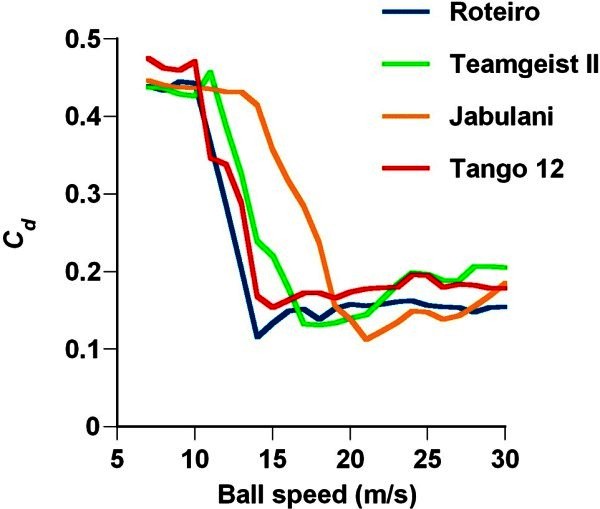
**Drag coefficient (*C***_***d***_**) of the Roteiro, Teamgeist II, Jabulani, and Tango 12.** The critical Reynolds number of the respective balls was ~2.2 × 10^5^ (*C*_*d*_ ≈ 0.12), ~2.8 × 10^5^ (*C*_*d*_ ≈ 0.13), ~3.3 × 10^5^ (*C*_*d*_ ≈ 0.11), and ~2.4 × 10^5^ (*C*_*d*_ ≈ 0.15).

The critical Reynolds number of the Roteiro, Teamgeist II, Jabulani, and Tango was ~2.2 × 10^5^ (*C*_*d*_ ≈ 0.12), ~2.8 × 10^5^ (*C*_*d*_ ≈ 0.13), ~3.3 × 10^5^ (*C*_*d*_ ≈ 0.13), and ~2.4 × 10^5^ (*C*_*d*_ ≈ 0.15) (Figure 4). The critical Reynolds number obtained for the Roteiro was the same as that reported by Asai et al. (2007). The standard *C*_*d*_ values for the Tango 12 and the Jabulani in the supercritical regime were ~0.18 and ~0.15, respectively. The average *C*_*d*_ in the subcritical regime was ~0.47, which was slightly larger than that of the Jabulani (~0.44). The newer balls showed an increased critical Reynolds number, and the *C*_*d*_ curve shifted to the right; however, the *C*_*d*_ curve of the Tango 12 was more similar to that of the Roteiro than to that of the Jabulani.

### Ball trajectory simulation

**Figure 5:**
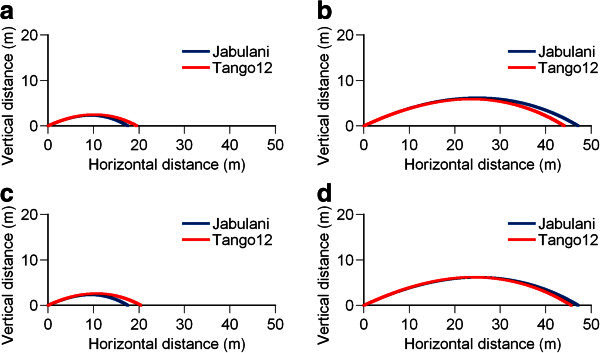
**Flight trajectory of the Jabulani and the Tango 12 in a simple 2D flight simulation.** (**a**) Initial ball velocity: 17 m/s. (**b**) Initial ball velocity: 28 m/s. (**c**) Impulse of ball impact: 7.45 kg/s. (**d**) Impulse of ball impact: 12.26 kg/s. Ball launching angle in all cases: 25°.

In the ball flight simulation, the flying distances of the Jabulani and the Tango 12 were respectively found to be 17.5 and 19.5 m for an initial velocity of 17 m/s and 47.1 and 44.1 m for an initial velocity of 28 m/s, with the ball launching angle being 25° in both cases (Figure 5). In simulations of ball impacts having the same impulse while considering the mass difference, the flying distances of the Jabulani (17 m/s) and the Tango 12 (17.4 m/s) for 7.45 kg/s were 17.5 and 20.4 m, respectively. Those of the Jabulani (28 m/s) and the Tango 12 (28.7 m/s) for 12.26 kg/s were 47.1 and 45.7 m, respectively.

### Extended total distances of panel bonds

**Figure 6:**
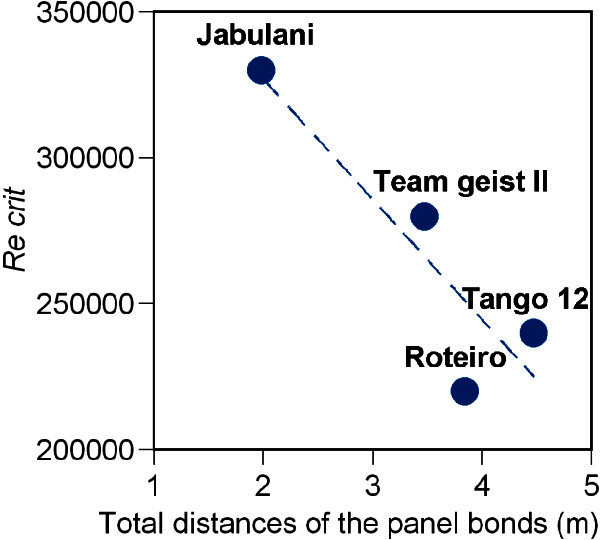
**Correlation between the extended total distances of the panel bonds and the critical Reynolds number (r = 0.9).**

The extended total distances of the panel bonds and the number of ball panels were as follows: Adidas Roteiro: 3840 mm, smooth surface with 32 pentagonal and hexagonal panels; Adidas Teamgeist II: 3470 mm, small protuberance with 14 panels; Adidas Jabulani: 1980 mm, small ridges or protrusions with 8 panels; and Adidas Tango 12: 4470 mm, small grip texture with 32 panels. High correlation was observed between the extended total distances of the panel bonds and the critical Reynolds number (r = 0.9) (Figure 6).

## Discussion

Achenbach (1972) claimed that the critical Reynolds number for a smooth sphere is ~3.5 × 10^5^, whereas Bearman and Harvey (1976) reported that the critical Reynolds number of a golf ball is ~6.0 × 10^4^. Therefore, it can be inferred that the critical Reynolds number of the soccer ball used in this study is lesser than that of a smooth sphere and greater than that of a golf ball. Because the Tango 12 has a smaller critical Reynolds number than the Jabulani, it is inferred that the former has lesser aerodynamic resistance than the latter in the medium-speed region (11 < *U* < 19 m/s), the near-critical region for the former. The former has greater aerodynamic resistance than the latter in the high-speed supercritical region (20 < *U* < 29 m/s).

In the flight trajectory simulation, the Tango 12 flew 2.0 m farther than the Jabulani when the initial velocity was 17 m/s, but it flew 3.0 m lesser when the initial velocity was 28 m/s. Similarly, in simulations with ball impacts having the same impulse (7.45 and 12.26 kg/s), the lighter Tango 12 flew farther. In the medium-speed region, where the coefficient of resistance of the Tango 12 was small, the difference in flying distances was as large as 2.9 m. However, in the high-speed region, where the coefficient of resistance of the Tango 12 was large, the difference in flying distances was reduced to 1.4 m.

These results suggest that the Tango 12, one of the newest soccer balls, has less air resistance in the medium-speed region than the Jabulani and can easily acquire large initial velocity in this region. In other words, this ball can easily gather speed in the frequently used medium-speed range, and therefore, it should be relatively suitable for a passing-based game of soccer.

The critical Reynolds number of each ball decreases with the number of panels, i.e. it decreases from the Roteiro to the Teamgeist II to the Jabulani. Furthermore, the extended total distance of the panel bonds decreases with the number of panels. The new Tango 12 ball has 32 panels; therefore, its extended total distance of panel bonds will increase, and its critical Reynolds number will be similar to that of the 32-panel Roteiro ball (r = 0.9). Achenbach (1974) reported that an increase in the roughness of the spherical surface decreases the critical Reynolds number. From these points, it can be concluded that the roughness of the ball surface increases with the extended total distance of panel bonds, causing the critical Reynolds number to decrease. In terms of roughness, the panel surface of the Roteiro is relatively smooth; the Teamgeist II has small protuberances; the Jabulani has small ridges; and the Tango 12 has small grip textures. Generally, the critical Reynolds number of a sphere decreases as the surface roughness increases. The critical Reynolds number of the Roteiro was lower than that of the Jabulani despite the panel surface of the Roteiro being relatively smoother than that of the Jabulani. The ‘small designs’ on the soccer ball panels appeared to play a small role in this experiment (see Additional file 1). Therefore, the critical Reynolds number of a soccer ball, as considered within the scope of this experiment, may depend on the extended total distance of the panel bonds rather than the small designs on the panel surfaces.

## Conclusion

This study aims to clarify the drag coefficient and critical Reynolds number of the newest soccer ball—the Adidas Tango 12 (32 panels)—and conventional soccer balls—the Adidas Roteiro (32 panels), Adidas Teamgeist II (14 panels), and Adidas Jabulani (8 panels)—through a wind tunnel experiment. Furthermore, a simple 2D flight trajectory simulation was conducted based on the drag coefficient, and the effect of the drag characteristics on the flight distance and flight trajectory of these balls was examined. The critical Reynolds number of the Roteiro, Teamgeist II, Jabulani, and Tango 12 was ~2.2 × 10^5^ (*C*_*d*_ ≈ 0.12), ~2.8 × 10^5^ (*C*_*d*_ ≈ 0.13), ~3.3 × 10^5^ (*C*_*d*_ ≈ 0.13), and ~2.4 × 10^5^ (*C*_*d*_ ≈ 0.15), respectively. The flight trajectory simulation suggests that the Tango 12, one of the newest soccer balls, has less air resistance in the medium-speed region than the Jabulani and can thus easily acquire large initial velocity in this region. The critical Reynolds number of a soccer ball, as considered within the scope of this experiment, may depend on the extended total distance of the panel bonds rather than the small designs on the panel surfaces.

## Electronic supplementary material

Additional file 1: **Drag coefficient (c) of Teamgeist (a) and Teamgeist II (b).** (JPEG 452 KB)
