# Supplementary figures and images for: Aerodynamic drag of modern soccer balls
Source: Springerplus. 2013 Apr 19;2:171. doi: 10.1186/2193-1801-2-171 (PMC3657093; doi:10.1186/2193-1801-2-171)

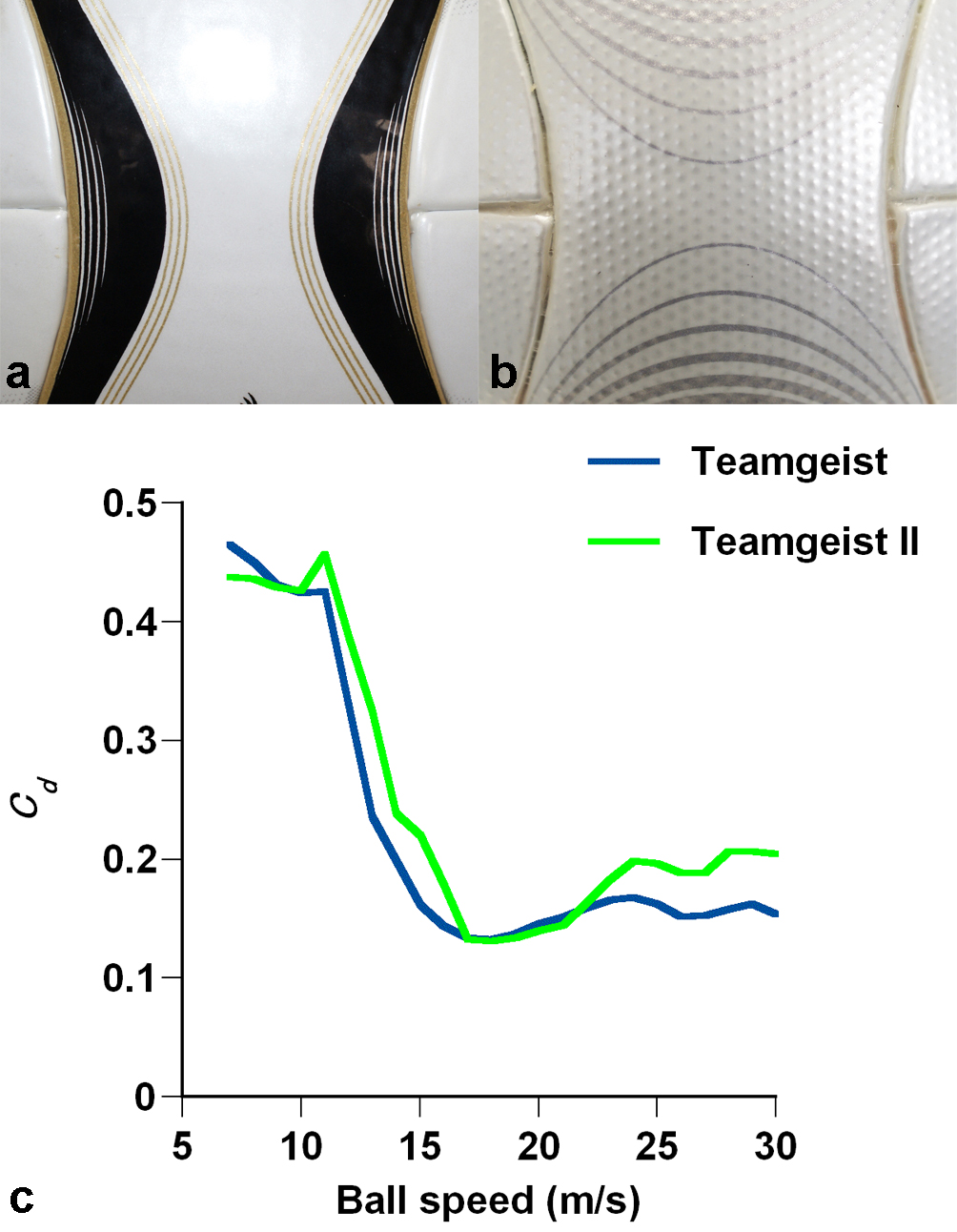

Supplement: Supplementary file 1 — Additional file 1: Drag coefficient (c) of Teamgeist (a) and Teamgeist II (b). (JPEG 452 KB) [file 40064_2013_261_MOESM1_ESM.jpeg]
